# Investment Strategies Used as Spectroscopy of Financial Markets Reveal New Stylized Facts

**DOI:** 10.1371/journal.pone.0024391

**Published:** 2011-09-14

**Authors:** Wei-Xing Zhou, Guo-Hua Mu, Wei Chen, Didier Sornette

**Affiliations:** 1 School of Business, East China University of Science and Technology, Shanghai, China; 2 School of Science, East China University of Science and Technology, Shanghai, China; 3 Research Center for Econophysics, East China University of Science and Technology, Shanghai, China; 4 Shenzhen Stock Exchange, Shenzhen, China; 5 Department of Management, Technology and Economics, ETH Zurich, Zurich, Switzerland; 6 Swiss Finance Institute, c/o University of Geneva, Geneva, Switzerland; Humboldt University, Germany

## Abstract

We propose a new set of stylized facts quantifying the structure of financial markets. The key idea is to study the combined structure of both investment strategies and prices in order to open a qualitatively new level of understanding of financial and economic markets. We study the detailed order flow on the Shenzhen Stock Exchange of China for the whole year of 2003. This enormous dataset allows us to compare (i) a closed national market (A-shares) with an international market (B-shares), (ii) individuals and institutions, and (iii) real traders to random strategies with respect to timing that share otherwise all other characteristics. We find in general that more trading results in smaller net return due to trading frictions, with the exception that the net return is independent of the trading frequency for A-share individual traders. We unveiled quantitative power laws with non-trivial exponents, that quantify the deterioration of performance with frequency and with holding period of the strategies used by traders. Random strategies are found to perform much better than real ones, both for winners and losers. Surprising large arbitrage opportunities exist, especially when using zero-intelligence strategies. This is a diagnostic of possible inefficiencies of these financial markets.

## Introduction

Nothing in biology makes sense except in the light of evolution. This famous sentence by Theodosius Dobzhanski [1] captures the fact that the extraordinary diversity of life can only be understood by combining the mechanisms of genetic evolution with historical environmental threads. Consider now the common wisdom that, as a result of accumulated technological and financial innovations, societal and economic networks have never been more complex and that this complexity has reached unmanageable levels within the current understanding and methodologies [2], [3], [4]. Moreover, this complexity is often accused to be at the core origin of the financial crisis that started in 2007, of the ensuing so-called Great Recession and of the continuing woes of major economies worldwide. In the spirit of Dobzhanski's statement, we here propose to investigate the concept that nothing in the complexity of financial markets make sense except in the light of the evolution of traders' strategies and of their mutual feedback loops. Standard approaches focus on “stylized facts” [5], [6], [7]. A stylized fact is a term used in economics to refer to a simplified presentation of empirical findings that are so consistent (for example, across a wide range of instruments, markets and time periods) that they are accepted as essential constraints that models or generating processes should strive to reproduce. Here, rather than fixating on stylized facts, we propose to study the combined evolution of financial patterns with the ecology of traders feeding on them and creating them. This is analogous to the importance of understanding the evolution of the fabric of social networks to make sense of the dynamics of human societies, the growth and organization of fault networks to account for the spatio-temporal organization of earthquakes, the structure of the brain and its plasticity to describe neural excitations and make progress on treating epileptic seizures, and so on. Similarly, the occurrence and severity of the financial crisis is best understood from the perspective of the accumulation of at least five bubbles over the last twenty years [8] associated with a climate of complacency everywhere and the illusion of the “great moderation” [9].

The view that financial markets can be better understood as adaptive ecologies of co-evolving traders is not new. It has been explored in agent-based models [10], [11], [12], [13], [14], [15] and articulated in the so-called “adaptive markets hypothesis” [16]. Here, our contribution is to provide novel empirical evidence based on the analysis of a unique dataset.

The logic of our approach is based on the following points.

1. Several important studies have shown that efficient allocation can result from the aggregation of decisions made by irrational or zero-intelligent agents under constraints [17], [18], [19].
2. Many studies have repeatedly documented that most traders underperform the global market as well as simple buy-and-hold strategies [20], [21], [22], with only very few exceptions [23].
3. The structure of markets results from the aggregate impact of traders.
4. Here, we show that random strategies are as a rule significantly better than even the best traders.
5. We characterize the statistical properties of trading frequency and holding periods of traders and quantify their impact on performance.

If most strategies were random, the conclusions are likely to be quite different. Points 3–5 together imply that there are untapped investment and arbitrage opportunities, that very few traders actually profit from. This is very surprising given the ease with which zero-intelligence traders over-perform. The quantitative characterization of the performances of traders as a function of a few key observable characteristics that is presented below can be used by future prospective traders to improve their strategies. Of course, as more traders become wiser, the market characteristics will evolve in a way similar to the first-entry games in which random strategies are found ultimately to dominate [24]. Our main point is that the characterization of financial markets requires understanding the ecology of strategies and their characteristics and how they interact together to shape the very patterns they exploit. Arguably, this will provide for really more efficient and robust financial markets, designed to avoid future systemic crises.

**Figure 1:**
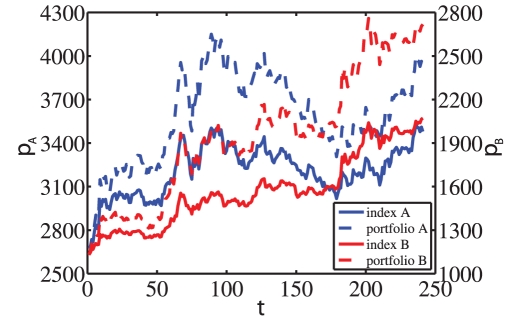
Time series. The daily evolution of Shenzhen Component indexes for A-shares and B-shares in 2003 and the performance of their 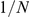 portfolios, respectively.

In this work, we perform a statistical analysis of the performance of all the traders trading 32 A-share stocks and 11 B-share stocks on the Shenzhen Stock Exchange of China in 2003. This market offers a unique opportunity to compare (i) a closed national market (A-shares) with an international market (B-shares), (ii) individuals and institutions and (iii) real traders to random strategies with respect to timing that share otherwise all other characteristics. The analysis is conducted separately for A-shares and B-shares. The database contains the information of each order including (i) the masked ID of the trader, (ii) whether he is an individual or institution, (iii) the direction, (iv) the price, (v) the size of the order, and (vi) the time stamps accurate to 0.01 second [25]. The evolution of the A-share index and the B-share index is shown in Fig. 1. Interestingly, the indices are found to be outperformed by the 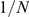 portfolio strategy [26]. We find that the net return of A-share individual traders is negative and independent of the trading frequency, while that of A-share institutional traders, B-share individual traders and B-share institutional traders decreases with increasing trading frequency. In addition, the net return decreases for winners and increases for losers when the trading frequency increases. We also find that random trading performs better for all individuals and institutions and for all winners. We show that the performance of traders exhibit non-trivial power law dependence as a function of trading frequency and holding periods.

### The invisible hand with zero-intelligence agents

Since Adam Smith's famous “invisible hand” description of economic and financial markets as self-regulating systems, economics has been dominated by the paradigm of rational utility maximizing agents. Given restrictive conditions, the agents' collective actions are found in theory to lead to stable general equilibrium points that are characterized by optimal allocation of resources. However, starting with H. Simon, and expanding with the work of D. Kahneman and A. Tversky as well as many other scientists, the severe limitations of human cognition and the many biases in real people's decisions have been pointed out. These limitations and biases a priori cast doubts on the relevance of rational utility theory. In reply, many studies have shown that irrational households can lead in aggregate to rational markets. In particular, Gode and Sunder [17] used “zero-intelligence” computer agents (who do not seek or maximize profits, do not observe, remember, or learn) to simulate market transactions in a double auction. They found that a population of such agents, subjected to budget constraint, produced results that closely mirrored the allocation efficiency of a simultaneous experimental human exchange. Studying prediction markets, Othman [18] confirmed recently that prices that replicate the findings of empirical market studies can emerge from a market populated by inhuman zero-intelligence agents with diffuse beliefs. Farmer et al. [19] developed a model of zero-intelligent agents that explains a large part of the cross-sectional properties of stocks traded in continuous double auction markets. They suggest that constraints imposed by market institutions may at times dominate strategic agent behavior, so that random agents with constraints perform on the whole as well as their more human siblings.

### Underperformance and the illusion of control

In both single-player and multiplayer Parrondo games, two or more losing games when alternated periodically or randomly yield a net winning outcome [27], [28], [29], [30]. When an optimization rule is introduced, the Parrondo games produce degraded rather than enhanced returns [31], [24]. This “illusion of control” phenomenon is present in other agent-based models whose design is inspired by stock markets [32]. The conventional wisdom states that institutions markedly outperform individuals because they are more informed [33]. However, the performance of both professionals and laymen is often documented to be worse than chance [34], [21], [22], [20]. Even worse, there is evidence showing that analysts' stock recommendation records are intentionally rewritten to a large extent [35]. Indeed, the performance of claimed successful strategies should be tested based on the method of random strategies, that are designed to remove survival and look-ahead biases [36].

The phenomenon of “Illusion of control” is one possible form of overconfidence. Overconfidence of stock market participants is expected to cause traders to trade more [37], [38], which has been confirmed at the market and individual equity level [39] and at the individual level [40], [41], [42]. In addition, there is evidence that the higher the frequency of trading, the poorer is the performance [43], [44], [33], [45].

## Materials and Methods

### Data sets

The Shenzhen Stock Exchange (SZSE) was established on December 1, 1990 and started its operations on July 3, 1991. It contains two independent markets, A-share market and B-share market. The former is composed of common stocks which are issued by mainland Chinese companies. It is opened only to domestic traders, and traded in CNY. The latter is also issued by mainland Chinese companies, while it is traded in *Hong Kong dollar* (HKD). It was restricted to foreign traders before February 19, 2001, and since then it has been opened to Chinese traders as well. At the end of 2003, there were 491 A-share stocks and 57 B-share stocks listed on the SZSE. In the year 2003, the opening call auction is held between 9:15 am and 9:25 am, followed by the cooling periods from 9:25 am to 9:30 am, and the continuous auction operating from 9:30 am to 11:30 am and 13:00 pm to 15:00 pm.

Our analysis is based on a database recording the order flows of 43 liquid stocks extracted from the A-share market and the B-share market on the SZSE in the whole year of 2003 when the close call auction was adopted in the opening procedure. The trading system did not show any information about the order flows, and traders submitted orders only according to the closing price of the last trading day. In the present work, we study a database in which, for each stock, all trading activities of all traders buying and selling the stock in the year 2003 are recorded. The database contains the price, size and associated time of each submitted order with the time stamps accurate to 0.01 second. Figure 1 shows the evolution of the two indexes.

### Method

Assume that there are 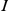 traders, each of them labeled 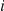, and there are 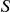 stocks in the database under investigation, each labeled 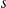. For trader 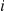 and stock 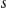, we can construct a sequence of buy/sell activities, denoted 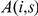:
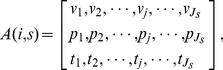
(1)which means that trader 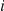 buys 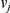 shares of stock 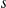 with price 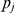 at time 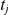 when 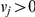 or trader 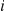 sells 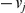 shares of stock 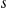 with price 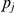 at time 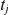 when 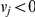. Obviously, there are no zero 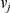 among all entries.

In order to construct 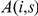, we need to reconstruct the order book. Assume that trader 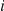 places a limit order of size 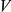 at time 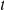, which is executed later by 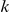 other effective market orders with sizes 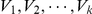 with prices 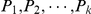 at times 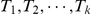. Then, we record only one entry 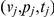, where
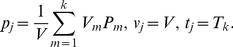
(2)This is very important in the calculation of transaction costs, defined soon. More operations are needed for 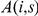 in order to make sure that
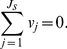
(3)First, if there are several sell transactions without any preceding buy transactions, these sells should not be included in 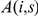. This is equivalent to treating the traders as new comers in 2003. Otherwise, we are unable to precisely calculate the earnings or the losses, since we do not know the price paid the trader when she entered the position at a time outside the year 2003, which is covered by our database. In the case of positions entered before 2003, if we would assume that the trader bought the stock right after the market opened on 2 January 2003, the calculated net return would be biased in general towards much higher values due to the rebound occurring around the beginning of 2003. Our results would be biased by the effective foresight given to the traders. In other words, this would correspond to giving the traders ex-ante forecasting abilities. We thus avoid such look-ahead bias [36] by removing sells without prior buys. Second, at the end of the year 2003, if trader 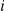 holds some shares of stock 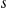, we added a new entry by including a fictitious transaction selling all his shares. This amounts to characterizing her position on a book-to-market basis at the end of our database.

This preprocess can be described in detail as follows. Assume that the initial array of transaction sizes for a trader trading a stock in 2003 is 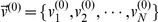, where 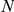 is the number of transactions. We then perform 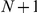 steps of operation. At the first step, if 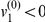 (for a sell), then 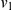 is discarded from 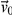 as discussed above, and the first element of the resulting array 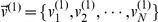 is updated and we have
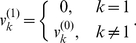
(4)Otherwise, if 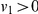, we have 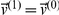. At the 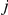
th step after there is at least one element 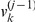 being positive for 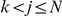, if 
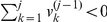
, we have
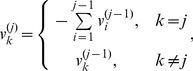
(5)which forms the updated array 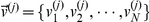. Otherwise, we have 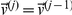. These 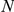 steps of operation is to ensure that
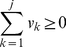
(6)for all 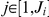. At the 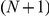
th step, if 
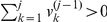
, we add a sell to the end of the array:
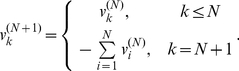
(7)To reduce computational time, we only need to perform these operations on sells. After discarding all zeros in 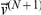, we obtain the first row of 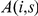.

For the 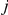-th transaction (or equivalently the 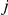-th entry), the trading volume is 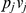. According to the Shenzhen Stock Exchange Trading Rules released in 2001, the transaction cost is determined as follows:
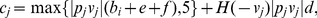
(8)where 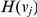 is the Heaviside function. The four terms in Eq. (8) are the following: (i) Brokerage 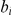, which should be less than 0.3%; (ii) Exchange fee 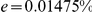 for A-shares and 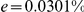 for B-shares for both buy and sell sides; (iii) Supervision fee 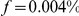 for both buy and sell sides; and (iv) Stamp duty 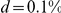 for sellers only (explaining the presence of the Heaviside function 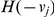 in expression (8)). The sum of 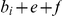 should be less than 0.3% with a minimum of 5 CNY for A-shares and 5 HKD for B-shares for both buy and sell sides. Note that 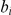 is 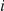-specific and independent of stock 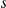.

Therefore, the total invested capital (the money that trader 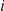 spent to buy stock 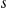) is 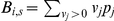 and the transaction cost of trader 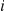 buying stock 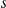 is 
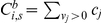
. The total capital obtained by selling all the shares of stock 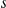 is 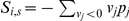 and the transaction cost of trader 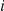 selling stock 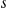 is 
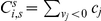
. The total transaction cost of trader 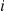 in his investment of stock 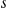 is
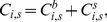
(9)The total earning is
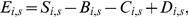
(10)where 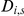 is the dividend received by agent 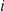 from stock 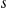 over the one period. The portfolio return of trader 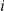 can be calculated as follows
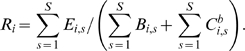
(11)The number of transactions (frequency of trading) is the sum of all 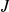 values of trader 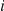


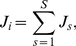
(12)where 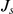 denotes how many times trader 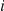 bought and sold stock 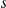, as implicitly defined in Eq. (1).

We note that the calculated invested capital in Eq. (11) could be larger than the real value, which will cause an underestimation of the absolute value of the return, although the net earning is accurate. However, it is hard to determine the true invested capital since we do not have any information about how much money the traders had in their accounts and whether they had added or withdrawn money from their accounts.

## Results

### Basic statistics

In our database, there are 2,330,093 A-share traders with 2,315,664 individuals and 135,086 B-share traders with 88,779 distinct individuals. It is found that the proportion of institutional traders is much higher in the B-share market (34.28%) than in the A-share market (0.62%). We choose to classify traders into “winners” and “losers” rather than “above-average” and “below-average”, or “upper half” and “lower half”, because we believe that this reflects the behavioral fact that traders care essentially about whether they are winning or losing money. Therefore, the statistics based on winners and losers are likely to better represent the distributions of results, as they reflect the natural target as well as metrics set by traders. For each trader 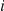, we calculate his portfolio return 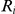. For A-share traders, 51.95% individuals and 68.50% institutions are net winners above the zero benchmark. For B-share traders, 85.76% individuals and 93.83% institutions are winners above the zero benchmark. These numbers are presented in Table 1.

**Table 1:** Numbers of all traders, winners and losers in the two markets.

	A-shares		B-shares	
	Individual	Institution	Individual	Institution
All traders	2,316,392	14,435	88,805	46,312
Winning traders	1,196,515	9,866	76,136	43,355
Losing traders	1,119,877	4,569	12,669	2,957

**Figure 2:**
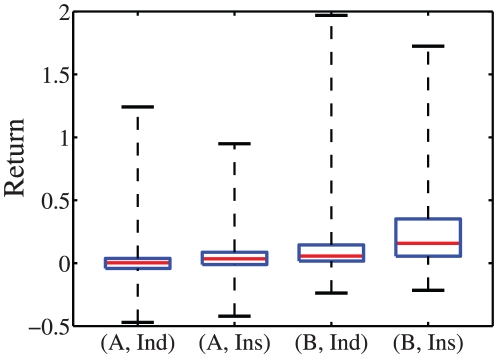
Box plots of the returns in four classes. The four plots are for individual A-share traders, institutional A-share traders, individual B-share traders, and institutional B-share traders. For each box plot, the minimum, lower quartile, median, upper quartile, and maximum of each class of returns are given.

To have a full understanding of how many of the traders are really performing, the box plots of returns for each type of traders in each market are given in Fig. 2. Some interesting observations are obtained.

- The proportion of winning traders in the B-share market is much higher than in the A-share market. This may be associated with the fact that the B-share index gained a much higher annual return than the A-share index (see Fig. 1). Passive or even under-performing agents perform better on an absolute basis, the higher the upward trend of the underlying market.
- In both markets, the winning proportion of institutional traders is much higher than retail traders, which is consistent with recent results found for the Taiwan market [33].
- In each market, individual traders may gain higher returns than institutions or incur greater losses when the performance is the best or the worst (see the maxima and minima in Fig. 2).

### Trading frequency and return

**Figure 3:**
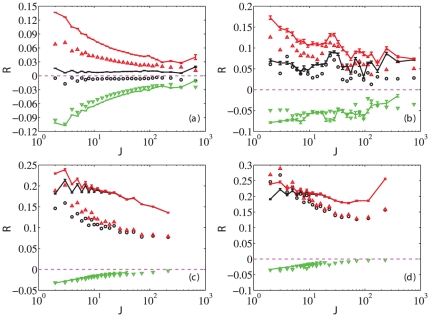
Dependence of the average return 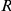
 **on the trading frequency** 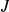 **.** Performance comparison of strategic trading (real data) and random trading using the average values of return 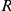 versus trading frequency 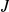. We exclude the sell transactions without any preceding matching buys. The simulations for the random strategies are repeated for 2000 times. We show the results for individuals in A-share (a), institutions in A-share (b), individuals in B-share (c) and institutions in B-share (d), respectively. In each plot, the colored symbols (black 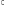 for all traders, red 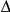 for winners and green 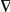 for losers) correspond to strategic trading, the continuous lines (black line for all traders, red line for winners and green line for losers) correspond to random trading, and the dashed line indicates the base line of zero return (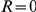).

Figure 3 shows the dependence of the average returns 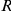 as a function of the trading frequency 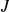 for A-share individuals (a), A-share institutions (b), B-share individuals (c), and B-share institutions (d), respectively. One can observe that the return is statistically independent of the trading frequency for A-share individuals while, in the three other cases, 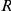 decreases systematically with 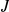, indicating that trading is hazardous to traders' wealth not only for individuals but also for institutions [44]. Since the numbers of institutional traders trading A-shares and B-Shares are relatively small, as shown in Table 1, the results for institutions have larger fluctuations. Moreover, the increase of 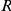 for large 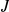 in Fig. 3(d) is not statistically significant since there were fewer foreign institutions conducting frequent transactions. Comparing the returns with the same trading frequency, institutions outperform individuals.

We also investigate the dependence of the net return as a function of trading frequency for two categories of traders, the winners and the losers. Winners (respectively losers) are defined as those having a positive (respectively negative) return. The classification is thus performed on an absolute (and not relative) basis. Figure 3 shows that return decreases with trading frequency for winners and increases for losers. The enhanced performance of losers by increasing trading frequency cannot be explained by a learned overconfidence bias as described in Refs. [37], [38].

Figure 3 also presents the average returns that random trading would yield in these markets. Random strategies are generated by considering each trader individually in turn, choosing random times for their trades while otherwise keeping fixed all other characteristics such as his number of transactions (trading frequency) and the trade sizes on each stock. Specifically, in Eq. (1), for a given trader 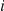 and a stock 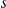, the variables 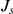 and 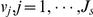 are unchanged, while the times 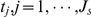 are replaced by a randomly chosen time sequence. As a result, the prices 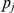 are also changed. This is done 2000 times for each trader, generating overall a very large number synthetic outputs contributed over all the traders in our database. For a given frequency 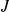, we sort again these many outputs into two classes: (i) the winners are the random strategies with a positive return; (ii) the losers are the random strategies with a negative return. We then compute separately the average returns (and their standard deviation) of the winning and of the losing random strategies, as well as the overall average return of these random strategies. Note that this construction of random strategies tests specifically the skills of traders with respect to timing, since all the other characteristics are kept otherwise identical [36]. According to Fig. 3, the aggregate net return of random trading (black lines) is higher than that of real trading (black circles) with the same trading frequency in every case. More impressively, the aggregate net returns of A-share individuals are negative, while the net returns using random trading strategy are positive. Two closely related conclusions can be drawn: (i) real trading is not random but strategic; (ii) the performance of strategic trading is worse than random trading. For the winners, random trading also induces higher return than real strategic trading in all four cases. For losers in the A-share market, random trading performs slightly worse. For losers in the B-share market, the random trading and real trading yield almost identical net returns.

### Holding time and return

**Figure 4:**
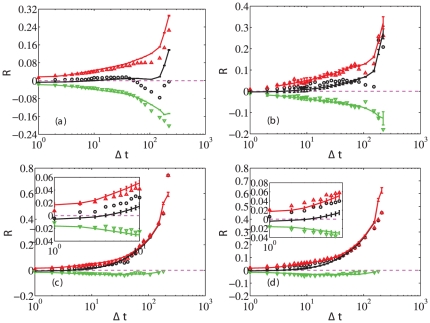
Dependence of the average return 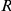
 **on the average holding time** 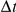 **.** Average returns 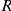 versus average holding time for individuals in A-share (a), institutions in A-share (b), individuals in B-share (c) and institutions in B-share (d), respectively. The symbols present the average values over all traders (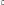), as well as traders who earn positive return (red 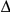) and negative return (green 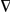). The dashed line delineates the benchmark in absolute terms of zero return. The solid lines with error bars are the average simulation results of random trading for all traders (black), winners (red) and losers (green). The insets in panels (c) and (d) are magnifications of the curves for small values of 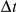.

We now study the influence of the holding time 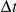, defined as the average of the elapsed time between the time a trader sold a stock and the time she bought it. In doing so, we need to identify buy/sell pairs. For each buy, an earliest sell with the same size is found to form a pair. If the size of the earliest sell is smaller than that of the buy, we need more sells to match the buy and the selling time is the average of all the associated time instants. The first row of 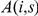 is used in this calculation. Figure 4 shows the average return 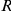 as a function of the average holding time 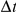 for A-share individuals (a), A-share institutions (b), B-share individuals (c) and B-share institutions (d), respectively. This figure is different from Fig. 3 because holding time is not simply the inverse of trading frequency. Indeed, the total holding time 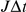 is not constant for different traders. In general, the aggregate net return increases with average holding time. This is consistent with the conventional wisdom that the buy-and-hold strategy outperforms most other strategies (see Fig. 1 for the performance of the buy-and-hold strategy of the 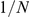 portfolio). For winners, the return is large when the holding time is long. For losers, 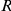 decreases with respect to 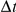 in the A-share market and varies slightly in the B-share market. The solid lines with error bars are the average simulation results of random trading. For large 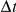 and B-shares, we still see that random trading performs better.

### Quantitative relations linking return to trading frequency and holding period

**Figure 5:**
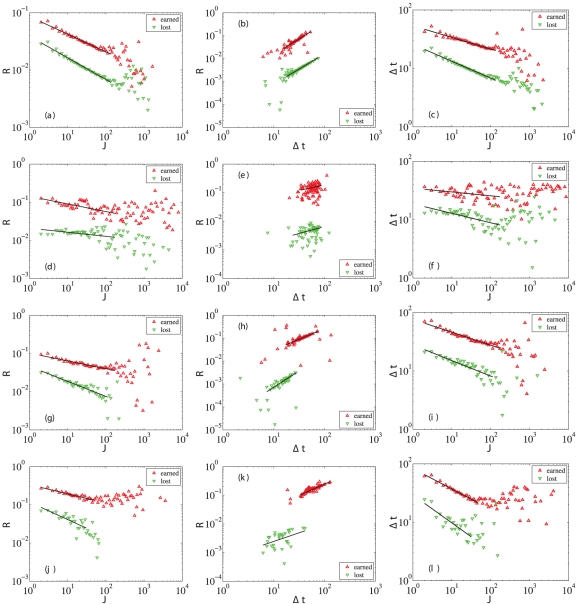
Power-law relationships between three variables. The first column (a,d,g,j) shows the dependence between the magnitude of the average return 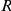 and the trading frequency 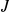. The second column (b,e,h,k) shows the dependence between the magnitude of the average return 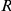 and the holding time 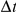. The third column (c,f,i,l) shows the dependence between the holding time 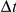 and the trading frequency 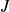. The four rows are for A-share individuals, A-share institutions, B-share individuals, and B-share institutions, respectively.

The winners or losers (individuals or institutions in a market) are sorted according to their trading frequencies. The averages of the returns and holding times of each group of traders are calculated. We plot the magnitude of the average returns for winners and for losers as a function of the trading frequency in double logarithmic coordinates in the first column of Fig. 5 and observe a power law relationship
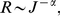
(13)where 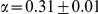 for A-share individual winners, 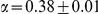 for A-share individual losers, 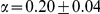 for A-share institutional winners, 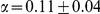 for A-share institutional losers, 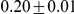 for B-share individual winners, 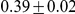 for B-share individual losers, 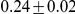 for B-share institutional winners, and 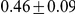 for B-share institutional losers.

The power law (13) and the obtained values of the exponents 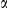 should be compared with the prediction from the simple null hypothesis that returns from the trading strategies are independent and identically distributed random variables with a finite variance (we have verified that this last condition holds in our data set). As the returns have approximately zero mean, as shown in Fig. 3a, the expected value of 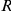 conditional on 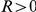 (or 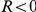) should be proportional to 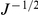 by the action of the Central Limit Theorem. Thus, the null hypothesis predicts a value 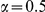. This value is strongly rejected by our measurements, which provides one-standard deviation error bars much smaller than the difference between 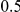 and the measured 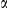
's. The significantly smaller measured values of 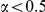 can be interpreted as due to the existence of persistency in the returns obtained by the trading strategies, the persistency being characterized by a Hurst exponent equal to 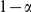.

Similarly, the magnitude of the average return 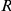 scales with respect to the average holding time 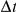 as a power law
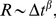
(14)and the average holding time 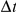 also scales with respect to the average trading frequency 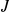 as a power law
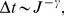
(15)as illustrated in the second and third columns of Fig. 5. The power laws are statistically more significant for individual traders than institutional traders because there are many more individuals (about 99.38%) in the A-share market. The estimated exponents are listed in Table 2. For each power-law relationship, the exponents for winners and losers are approximately equal to each other, that is,
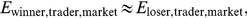
(16)where 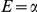, 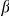, or 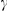,“trader” could be individuals or institutions, and “market” could be A-shares or B-shares.

**Table 2:** Estimated exponents of the power-law relationships.

	A-shares				B-shares			
	Individual		Institution		Individual		Institution	
	Winner	Loser	Winner	Loser	Winner	Loser	Winner	Loser
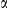	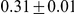	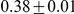	**0.20** 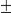 **0.04**	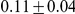	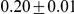	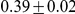	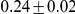	**0.46** 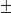 **0.09**
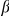	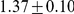	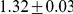	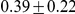	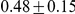	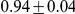	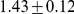	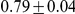	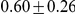
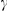	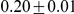	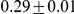	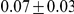	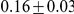	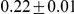	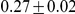	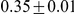	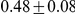
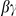	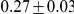	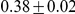	**0.03** 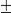 **0.03**	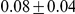	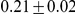	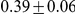	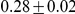	**0.29** 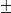 **0.17**

Combining Eqs. (13–15), we obtain an equation relating the three power-law exponents
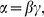
(17)which is validated in Table 2 for most cases. A careful examination shows that this relation between exponents does not hold in the two cases given in Table 2 that are marked in bold face. These two cases correspond to institutional traders. This discrepancy is caused by the relatively smaller numbers of institutional traders in both the A-share and B-Share markets, as shown in Table 1. Indeed, the agreement is much better for individual traders than for institutional traders since there are many more individual traders.

## Discussion

The original incentive of this study was to provide novel comparative characterizations of financial markets that are based on the realized performances of strategies implemented by traders. The Shenzhen Stock Exchange of China offers a unique opportunity to compare (i) a closed national market (A-shares) with an international market (B-Shares), (ii) individuals and institutions and (iii) real traders to random strategies with respect to timing that share otherwise all other characteristics.

The first robust result is that more trading results in smaller net return due to trading frictions. This is true for both individual and institutional traders in China's B-share market. However, the net return of individual traders in the A-share market is independent of the trading frequency, which is different from other markets [43], [44], [33], [45]. For individual or institutional winners, this result holds again. We unveiled quantitative laws showing how the deterioration of performance scales with frequency and with holding period. Naively, we could have expected that the performance is simply inversely proportional to the trading frequency, if transaction costs was the only contribution. But here, we find non-trivial exponents, which reveal the complexity of the market price structure as the traders strategically adapt their investments. The complexity of the market price structure is partially quantified by the persistence in the strategy performance as well as in the holding time of positions, characterized by the exponents 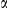 and 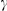 defined by expressions (13) and (15). These results provide a new set of stylized facts that characterize the structure of the price patterns. In other words, the properties of the returns obtained by different traders provide a kind of “spectroscopy” of the prices. By the term “spectroscop”, we use the analogy with the physical technique with the same name, defined as the study of the interaction between matter and waves. In the physical sense of the term, in many applications, spectroscopy uses the interaction between matter and wave to probe the former. It is in that sense that we propose that the study of the performance of trading strategies provide new probes and novel understanding of financial price time series.

We also found that the return of real trading is significantly and robustly worse than random trading. As a consequence, we can conclude that traders do try to develop opportunistic strategies, but zero intelligence strategies outperform them in stock trading. Certainly, this conclusion does not deny the possibility that some traders do perform better than random trading. Therefore, we can use the strategy performance as a gauge or an instrument to characterize the market structure, in addition to the its statistical properties often referred to as the stylized facts. To the best of our knowledge, this idea is novel. It reflects the natural consequence that the aggregation of strategies make the stock market structure what it is, and vice-versa the later influences and co-evolve with the ecology of strategies [13]. The strategies implemented by traders are not only probing the prices but also influencing the prices so that they are both cameras and engines [46]. We believe that the study of the combined structure of both strategies and prices will open a qualitatively new level of understanding of financial and economic markets.
